# Temporal approach to identifying ectomycorrhizal community associated with Mongolian pine in a desert environment, northern China

**DOI:** 10.1128/spectrum.02026-23

**Published:** 2023-09-14

**Authors:** Yue Ren, Guanglei Gao, Guodong Ding, Ying Zhang, Peishan Zhao, Jiayuan Wang

**Affiliations:** 1 Yanchi Research Station, School of Soil and Water Conservation, Beijing Forestry University, Beijing, China; 2 Key Laboratory of State Forestry and Grassland Administration on Soil and Water Conservation, Beijing Forestry University, Beijing, China; 3 State Key Laboratory of Efficient Production of Forest Resources, Beijing Forestry University, Beijing, China; 4 Engineering Research Center of Forestry Ecological Engineering, Ministry of Education, Beijing Forestry University, Beijing, China; Broad Institute, Cambridge, Massachusetts, USA

**Keywords:** ectomycorrhizal fungi, fungal diversity and community structure, temporal variation, climate, Mongolian pine

## Abstract

To date, the ectomycorrhizal (EM) fungi community has been widely investigated with aging plantations affected by the pedologic factors. However, knowledge on the effects of phenology induced by climatic factors across the age range remains poorly understood on an intra-annual scale. Here, we sampled the fine roots of Mongolian pine (*Pinus sylvestris* var. *mongolica*) plantations at different stages of the growing season (from April to September) across three stand ages (27, 34, and 44 yr) in the Mu Us Desert, Northwest China. We aim to disentangle the community composition and structure of EM fungi, as well as the impact of climate on EM fungi. We observed that the 173 distinct EM fungal operational taxonomic units (OTUs) were identified. *Geopora*, *Inocybe*, *Tomentella,* and *Tuber* were the most frequent, and their dominance was maintained as stand aging. The richness and community composition were not significantly different with stand aging (*P* > 0.05). Host phenology and stand age are two important factors that have shaped the EM fungal community. The growing stage affected the beta diversity of the EM fungal community more than stand age, and this variation of the EM fungal community was closely related to seasonal climate, particularly precipitation. This improved information will provide a theoretical basis for the reforestation and rehabilitation of the Mongolian pine plantations using mycorrhizal techniques.

**IMPORTANCE:**

Ectomycorrhizal (EM) fungi are particularly important for host plants in a desert ecosystem. With a high degree of plasticity, EM fungi are largely influenced by host plant and environmental variables and fundamentally contribute to the ability of individuals to adapt to environmental changes. Therefore, the EM fungi are important for Mongolian pine (*Pinus sylvestris* var. *mongolica*) plantation in a desert ecosystem. Although previous studies have concluded that multiple endogenous and exogenous processes ultimately lead to species-specific temporal patterns in EM fungal populations. We still neglect the effect of host phenology on EM fungal activity. The significance of our study is the interplay between climate-driven EM fungi and plant phenology.

## INTRODUCTION

As a vital mediator between soil and host plant, ectomycorrhizal (EM) fungi infect tree roots and form symbiotic relationships, which is an important functional group in the soil system (1, 2). EM fungi rely on host plants for nutrients, and in return they contribute to the mineral nutrients and water utilization of host plants to resist environmental stress through the extraradical mycelium (3, 4). Therefore, EM fungi are essential for plant nutrient transport, biodiversity maintenance, and ecosystem stability across temperate and boreal forests ecosystem (5, 6).

EM fungal community is complex with a high diversity and variability at multiscale (7), affected by a series of abiotic (e.g., elevation, climate, and soil property) and biotic factors (e.g., stand age and phenological stage), as well as the interspecific interactions of the fungi (8 – 10). Host preference of EM fungi drives the variations in community composition and structure (11). Climatic condition is recognized as a crucial driver for the variations in soil fungal communities (12), which affects mycorrhizal formation and development (13), and thus the dynamics of fungal diversity, species richness, and community compositions (10, 14, 15). Consequently, climate apparently has strong impacts on local and even regional scales. Furthermore, it has been shown explicitly in the previous study that the edaphic variable is a momentous constituent of the EM fungal community at the rhizosphere (16). Changes in soil properties can indirectly affect EM fungal communities and succession via plant growth and stand structures (17). pH and N deposition induce soil nutrient imbalance and profoundly cause fungal competition for resources to accommodate nutrient cycle processes (18). While climate and soil conditions have largely accounted for the heterogeneity of EM fungal communities, more importantly, host preference or specificity of EM fungi is credited with driving variability in community richness and composition (1, 11). With the development of forest stands, host plants modify soil properties through plant biomass and litter input, exudation of allelopathic in the rhizosphere, consumption of soil water and nutrients, and soil microclimate (6). All mentioned may differentially affect the competitive ability of EM fungi (18). The host phenology affects the EM fungal activity through seasonal photosynthetic allocation and carbohydrate availability of roots (19), thus driving a general intra-annual pattern of fundamental functional groups of the EM fungi (20). To sum up, a variety of endogenous and exogenous processes eventually lead to species-specific temporal patterns in EM fungal populations. Therefore, knowledge of the temporal dynamics of EM fungal communities is key to understanding fungal diversity and ecosystem functioning.

The Mu Us Desert is located in the semi-arid region of northern China, with severe water shortages, and highly seasonal temperature and precipitation. Low nutrient availability, high temperatures, and water scarcity constrain plant growth and productivity in arid and semi-arid ecosystems (21 – 23). Many desert plant species are dependent on symbiotic relationships with EM fungi for efficient nutrient and water acquisition (24, 25). As a result, variations in precipitation and soil moisture fluctuate in response to seasonality and subsequently induce changes in the community structure of EM fungi in desert regions. The spatial structure of desert EM fungal communities has been addressed in several recent studies (7, 26). These studies have identified deterministic (e.g., edaphic conditions, including soil temperature, moisture, and nutrient availability) as well as dispersal limitation as important determinants of fungal biomass, community composition, and species richness on fine and broader spatial scales. However, the importance of plant phenology in desert habitats has been overlooked.

Mongolian pine (*Pinus sylvestris* var. *mongolica*) is a strongly cold-resistant and drought-resistant shelterbelt tree species and widespread reforestation in northern China (27, 28). Mongolian pine was first planted for sand fixation in the Horqin Desert in the 1950s, and science then it has been introduced to more than 13 provinces in desert regions (29, 30). Up to the present, the total area of the Mongolian pine plantation has reached more than 7.00 × 10^5^ ha, which greatly contributes to desertification control and degraded land restoration (31). However, severe degradation in Mongolian pine plantations has occurred since the mid-1990s (32). As an ectomycorrhizal-dependent species, EM fungi are of great significance to the Mongolian pine plantation. Gradually, soil microbes have raised serious attention and proposed that soil microbes are the impalpable but unavoidable driving factors (33). Previous studies have shown that the loss of key functional root-associated fungi (especially EM fungi) is perhaps an important factor in the degradation of the Mongolian pine plantation (28, 33, 34). Therefore, understanding the interplay between EM fungi and seasonal variations in phenology is critical for sustainable forest management in climate- and environmental-sensitive zones.

To this end, we have studied the temporal variations in the EM fungal community structure associated with Mongolian pine plantations and predicted the relationship between host phenology, stand age, and EM fungal community. Here, we set up two sets of temporal gradients, one based on the long-term variation of the stand age and the other based on the short-term temporal/seasonal variation of the month. It was therefore hypothesized that (i) EM fungal composition and species diversity would exhibit distinct temporal variations and (ii) phenological stage rather than stand age determines the EM fungal community composition.

## RESULTS

### Community diversity of EM fungi associated with Mongolian pine

In total, 1,816,286 high-quality sequences were obtained from all the root samples, and they were divided into 2,609 fungal operation taxonomic units (OTUs). After that, the quality sequences were clustered into 173 EM fungal OTUs at a sequence similarity threshold of 97% (Table S1). All EM fungal OTUs were assigned to 2 phyla, 3 classes, 12 orders, 28 families, and 38 genera. The number of EM fungal OTUs was highest in July, only a few OTUs were common in all growing seasons (Fig. S1). The numbers of the unique EM fungal OTUs were the highest in MUn, and there were 84 OTUs shared by the three stand ages (Fig. S1).

**Fig 1:**
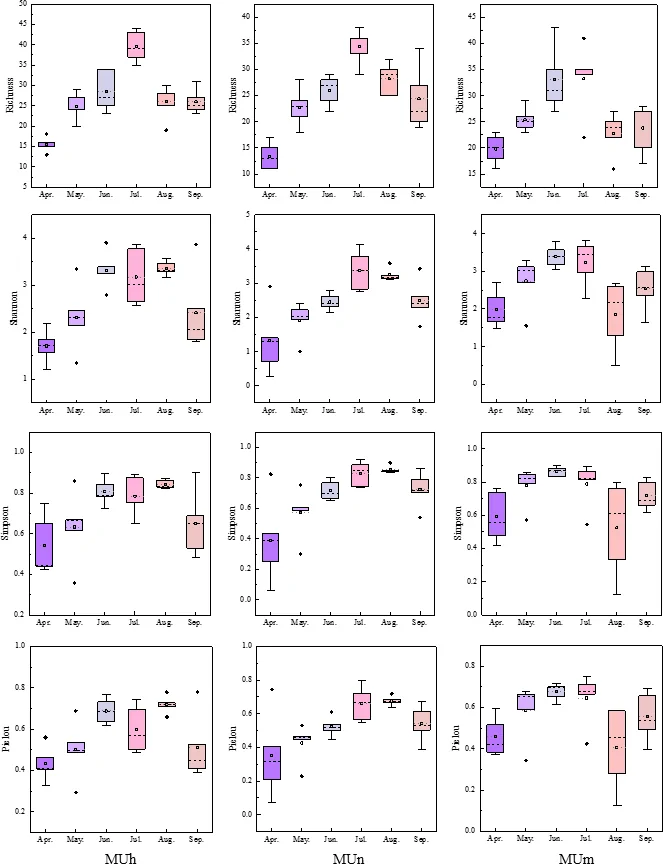
Diversity indices of EM fungi at different stages of growing season across three stand ages (MUh, half-mature; MUn, nearly mature; MUm, mature). The index values at each age group are averaged by month (April, May, June, July, August, and September).

The growing season had a significant impact on the EM fungal diversity (*P* < 0.01, Table S3). The maximum value of alpha diversity indices appeared in July, while the minimum diversity indices appeared in April (Fig. 1). The effect of stand age on EM fungal diversity was not significant (*P* > 0.05, Table S4), the value of it decreased first and then increased with stand aging, with the highest value for MUh. The variations of phenological month rather than stand age had the largest temporal difference in the EM fungal diversity.

### Community composition of EM fungi associated with Mongolian pine

**Fig 2:**
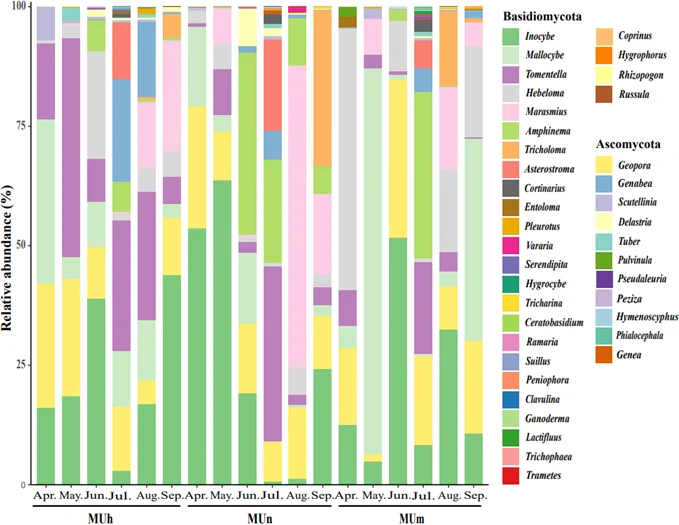
The relative abundance of EM fungi at different stages of growing season across three stand ages at the genus level.

Basidiomycota was the dominant phylum (75.14% of total OTUs), with Agaricales (76 OTUs) being the dominant order. In Ascomycota (24.86%), the order Pezizales (38 OTUs) was the most prevalent. The relative abundance of Basidiomycota gradually decreased with stand age and phenological month. A distinctive EM fungal community structure was observed for all Mongolian pine age groups in different months (Fig. 2). *Inocybe, Geopore, Mallocybe,* and *Tomentella* maintained a relatively high frequency throughout all sampling months, with a mean relative abundance of 23.30%, 15.21%, 13.63%, and 12.23%, respectively. In addition, *Hebeloma*, *Marasmius*, *Amphinema*, *Tricholoma*, *Genabea*, and *Asterostroma* were prevalent at different stages of the growing season across three age groups. *Tomentella* was more abundant in May and July, *Inocybe* had a higher proportion in June and September, and *Mallocybe* dominated in April. The EM fungal genera were widely distributed in July. *Inocybe* was abundant in various age groups.

### Temporal difference in composition and structure of EM fungi associated with Mongolian pine

**Fig 3:**
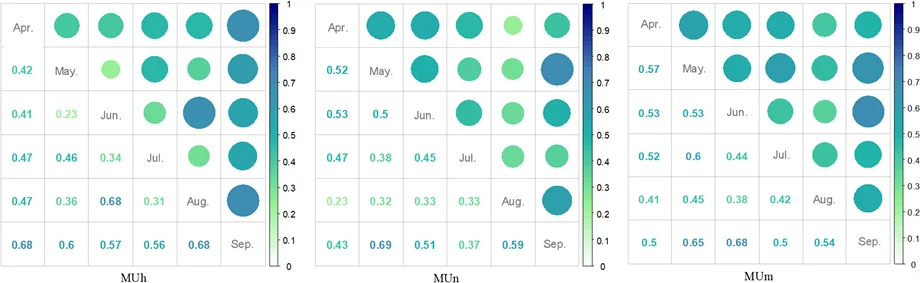
Community composition similarity of EM fungi at different stages of growing season across three stand ages. Blue indicates positive correlation, and green indicates negative correlation. The numbers in the figure denote the degree of community similarity. Larger circles and numbers indicate higher community similarity.

There was a diverse similarity of EM fungal composition associated with Mongolian pine at different stages of the growing season across three stand ages (Fig. 3). In MUh, the similarity between adjacent months was worse, except in August and September. In MUn, the similarity of EM fungal composition was worse in July and August. In MUm, the taxonomic community composition and structure were more similar between different months. In general, the similarity degree of EM fungal composition was better between July, August, and September but worse between July and other months.

**Fig 4:**
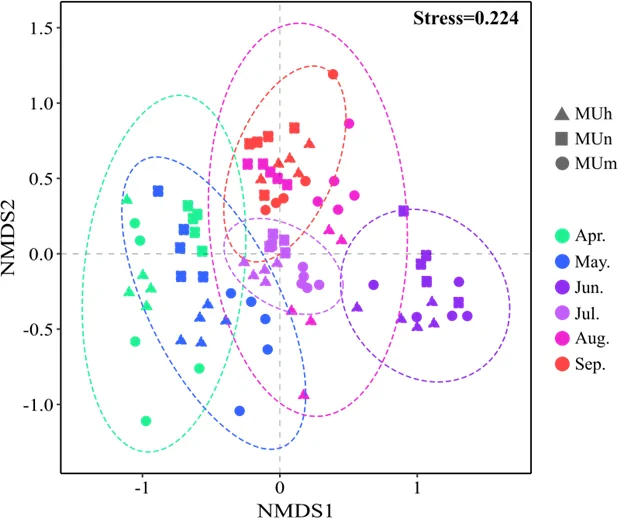
Ordination of EM fungal communities at different stages of the growing season across three stand ages based on OTUs, as determined on NMDS using the Bray-Curtis distance. Stress = 0.224. Different shapes in the figure represent different age groups, and different color points represent different months.

The distribution of the EM fungal communities in different stages of the growing season across three age groups was visualized using non-metric multidimensional scaling (NMDS) based on the Bray-Curtis dissimilarity index (Fig. 4), which showed that the EM fungal community composition was obviously separated by the stage of growing season (PERMANOVA: *F* = 6.202, *P* = 0.001) and stand age (PERMANOVA: *F* = 3.063, *P* = 0.001). The growing season has a stronger effect on the EM fungal community structure (Table S3). Shift in EM fungal community structure was closely related to climate factors and month, especially precipitation (Table S5). However, after accounting for these factors, the effect of stand age became non-significant.

### Keystone OTUs and co-occurrence network of EM fungi associated with Mongolian pine

**Fig 5:**
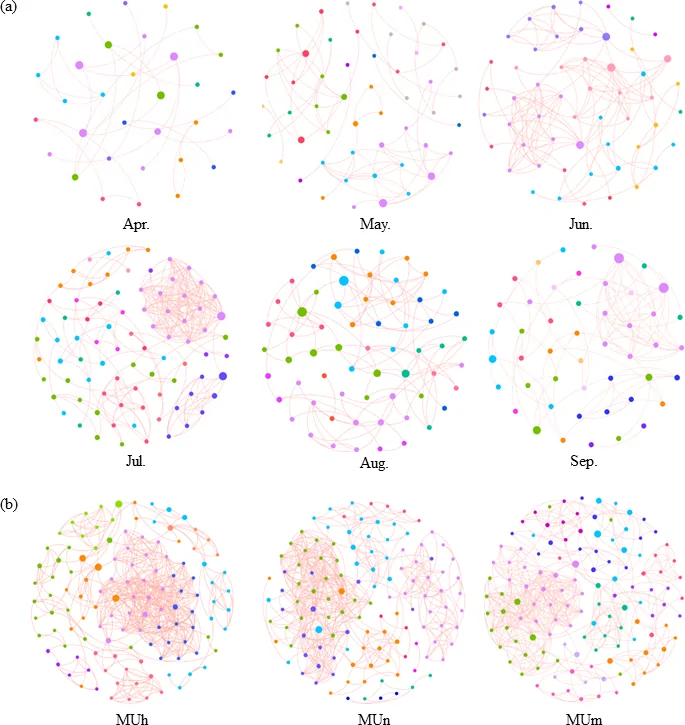
Co-occurrence networks of the EM fungi associated with Mongolian pine at different stages of the growing season (a) across three stand ages (b). Node size is the betweenness centrality of each OTU, colors represent different modules. Pink edges represent positive correlations.

Nine co-occurrence networks of EM fungi at different stages of the growing season across three stand ages were constructed, separately (Fig. 5). All networks are modular (modularity: >0.4), and all network properties imply the typical small-world characteristic of the nine empirical networks. Network complexity varied considerably across the different stages of the growing season and exhibited temporal variations. The network characteristics in July were more complex than the other growing seasons, with more nodes and edges (83 and 246) and less modularity (Table 1). Relative to the stage of the growing season, modularity varied less among three stand age networks of EM fungal communities. Consequently, our results suggested that the variation across the stages of the growing season was stronger than that across stand ages (Table 1).

**TABLE 1:** Topological features of the co-occurrence networks of EM fungal communities at different stages of growing season across three stand ages^*a*^

Topological features		Month						Age group		
		April	May	June	July	August	September	MUh	MUn	MUm
Empirical network	Node	32	51	53	83	62	48	121	123	125
	Edge	31	69	128	246	102	90	507	479	473
	Modularity	0.804	0.853	0.724	0.639	0.826	0.683	0.619	0.679	0.685
	Average degree	1.938	2.706	4.830	5.928	3.290	3.750	8.380	7.789	7.568
	Average path length	3.146	1.309	2.964	2.208	2.853	1.295	6.651	3.591	6.601
	Average clustering coefficient	0.667	0.860	0.774	0.790	0.745	0.913	0.716	0.689	0.732
	Positive	31 (100%)	69 (100%)	128 (100%)	246 (100%)	102 (100%)	48 (100%)	31 (100%)	69 (100%)	31 (100%)
	Negative	0	0	0	0	0	0	0	0	0
Random network	Average path length	2.501 ± 0.064	2.339 ± 0.041	2.223 ± 0.027	2.065 ± 0.021	2.126 ± 0.023	2.165 ± 0.031	1.940 ± 0.013	1.946 ± 0.017	1.936 ± 0.014
	Average clustering coefficient	0.081 ± 0.025	0.110 ± 0.02	0.085 ± 0.017	0.090 ± 0.015	0.091 ± 0.015	0.098 ± 0.019	0.238 ± 0.015	0.231 ± 0.017	0.235 ± 0.016

^*a*^ Random networks were generated by rewiring all of the links of a corresponding empirical network with identical nodes and links. Correlation: number of edges (proportion).

**Fig 6:**
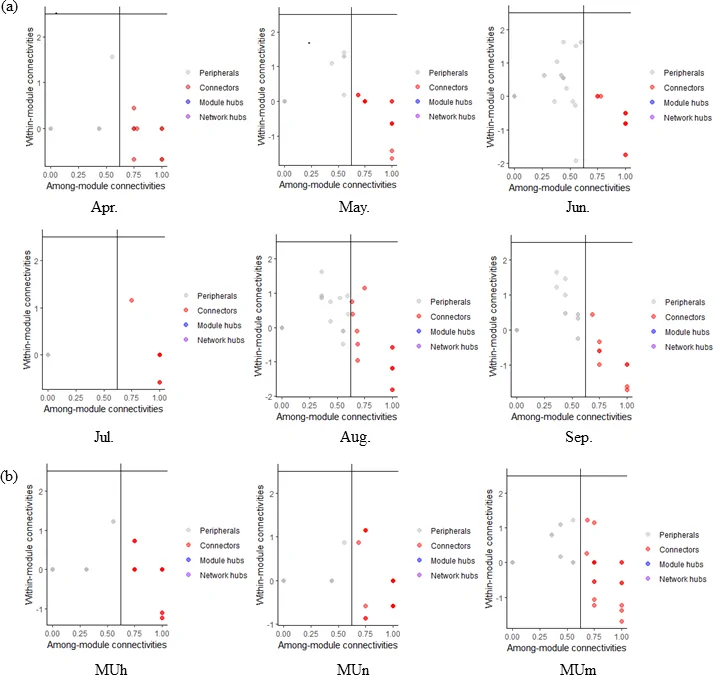
Keystone OTUs of EM fungi associated with Mongolian pine at different stages of the growing season (a) across three stand ages (b).

The keystone OTUs at each growing season were different. No module hubs were detected in any of the networks. Only a few nodes were designated as “Connectors” in each growing stage network (Fig. 6). We found that more connectors appeared in August and MUn (Fig. 6). During the vegetative growing phase in the beginning stage (April), members of Basidiomycota were the most prominent keystone taxa in the EM fungal network. By the stage of leaf flush (May), Ascomycota has become minor component of the connectors. *Inocybe* and *Tomentella* were prominent connectors of the modules at all growing stages. Moreover, some OTUs acted as a keystone for multiple networks (Table S5). OTU324 (*Hebeloma mesophaeum*) was classified as connectors in four different vegetative phases (Table S6).

## DISCUSSION

### Temporal variations in EM fungal diversity

As a huge underground ecosystem, forest microorganism has diversity and heterogeneity (35). Vegetative growing season contributed substantially to the determination of the EM fungal assemblage, the communities developed over time as plants grew. Variations in the diversity index of EM fungi according to stand age were inconsistent across the different stages of the growing season. Previous studies on the relationship between EM fungal diversity and stand age also revealed that there was a clear relationship between EM fungal diversity and host status (36). We expected a higher abundance of the EM fungi in mature forests, as many authors have noted a lower-diversity EM fungal community in younger forests, which increases with the root system and stand development (37). However, the EM fungal species in half-mature forests were the most abundant, this outcome of the EM fungal community comparison associated with Mongolian pine plantation was just the opposite as expected. The answer is many fungi that existed in nearly mature forests disappeared in mature forests during stand development, although their abundance and dominance changed within a year. Differential richness and diversity of the EM fungi among the stage of the growing seasons were attributed to contrasting patterns in different growth phenology. In Mu Us Desert, the effect of growing stage on fungal diversity was significantly stronger than stand age. EM fungal communities varied significantly by month, under the influence of monthly precipitation and mean temperature. We found that the number of EM-OTUs was significantly higher in July, August, and September than those in April, May, and June. This finding was consistent with previous results as soil moisture is affected by precipitation and temperature and correlated with fungal community composition (34). It was also found that low precipitation suggests a relatively low fungal diversity, especially in desert areas (38). Serious drought is likely to enable higher mycelial production, resulting in intensified competition for space (20, 39). In many of the temperate forest regions, precipitation concentrated in the summer is common. Also, some studies reported seasonal shifts in EM fungi with maximum abundance in summer (9) or autumn (40). The ectomycorrhizal associations also occur in regions with abundant soil moisture. In addition, the decay of soil extramatrical mycelia reflects an annual cycle of the EM fungi in temperate ecosystems, which shows increasing diversity and biomass during the summer (3). Because of the foregoing, it is not surprising that the richness and diversity of EM fungi in July are higher than that in other months.

### Temporal variations in EM fungal community structure

Ectomycorrhiza on Mongolian pine has been frequently studied in recent years (7, 28, 34). The abundant EM fungal OTUs identified in Mu Us Desert are generally known as common members of fungal communities of temperate and boreal forests, and they have also been identified previously in other Pinaceae tree species (41), including members of the genera *Geopora*, *Inocybe*, *Rhizogopon, Tomentella*, etc. Besides, genera *Tuber*, *Amanita*, *Boletus*, *Sebacina*, *Hebeloma,* and *Amphinema* were already found to be associated with and typical in *P. sylvestris* forests. In the Mu Us Desert, EM fungal community associated with Mongolian pine was mainly represented by *Geopora*, *Inocybe,* and *Tomentella*, which is consistent with other reports on Mongolian pine fungal communities (30, 33). Although previous researches on EM fungi have emphasized the importance of fungi in Basidiomycota due to their abundant production of epigeous sporocarps. However, research in recent decades indicates that members of the Ascomycota are important in many habitats (42 – 44), including environmentally stressful conditions such as drought forests (45). The largest genus of Basidiomycota was *Inocybe*, and it has a considerable relative abundance in different sampling months and stand ages. The abundance of *Inocybe* may be explained either by its advantageous competition ability (46), especially under arid conditions (47). *Inocybe* is ecologically important in forest succession by providing water and minerals for the host plants (48). The relative abundance of *Rhizopogon* in mature forests was higher, which was consistent with previous research (28). Because they are typical “late-stage” fungi with a high demand for carbohydrates (49). Many Ascomycota EM fungi belong to the Pezizales. It has been reported that EM fungi within the order Pezizales were well adapted to harsh environments and regenerated quickly after disturbance (50). In addition, dominance by Pezizales has been observed on plants growing in low moisture and nutrient soils (51). The OTUs with the highest abundance among the identified root-associated Pezizales were *Geopora*. Species of *Geopora* are important ectomycorrhizal with a wide geographical range and also involved diverse host plants including coniferous and deciduous, as well as orchids. Moreover, *Geopora* species were considered to be an important mutual partner for host plants resisting the stress conditions due to the abundance in the forest with drought and barren soil conditions (44).

EM fungal community composition associated with Mongolian pine at different stages of the growing season was diverse with various genera in the Mu Us Desert. But in general, EM fungal composition was composed of dominant Basidiomycetes and a few Ascomycetes, which is consistent with general EM fungal distribution (52). EM fungal community composition has clear temporal differences due to seasonally variable environmental conditions in desert regions. This temporal variation of the EM fungal communities is expected because of individual differences in tolerance to high temperature and low moisture among fungal species (53). As a better competitor for space, Basidiomycetes would maximize the efficiency using the exploit available resources and convert them to biomass (54). In this study, the differences in Ascomycetes fungi were not statistically significant, partly as a result of the low number of OTUs and the non-substantial spatial variation. We found a lower frequency of Ascomycetes fungi in middle-aged forests, as it has a survival strategy that increased abundance in poor-nutrient and more acidic environments. Significant differences in the EM fungal community between stand ages were also reflected in the abundance of EM fungi at the genus level. The higher richness of EM fungal OTUs that we found in middle-aged forests may be due to the availability of photosynthates and C allocation to roots, which may become active earlier. In association with middle-aged forests, Boletales dominate, with a significant difference from mature forests. However, there was no significant difference in fungal community structure across different stand ages. There was a protective and feedback interaction between the host root system and EM fungi, which is relatively stable (10), while the root micro-environment provides a stable habitat for EM fungi in host plants’ roots through a strong environmental filter (55). Moreover, the low percentages of *Wilcoxina* and *Suillus* compared with previous studies in the Mu Us Desert (7, 33, 36) suggest that Mongolian pine plantations in this region suffer from an acute deterioration process or perturbation, probably as a consequence of human and livestock activity and environmental degradation.

### Interactions between EM fungal community composition, climate, host phenology, and stand aging

In forest ecosystems, the correlation of aboveground plant phenological characteristics with belowground root-associated fungal communities has rarely been studied, due to the complex interactions in the host and soil microbial. Our results have demonstrated that EM fungal community in temperate forest soil is very dynamic, showing significant temporal variations in composition and relative abundance of different fungi. During stand aging, some less competitive fungi appeared in the early stage, while some highly competitive fungi persisted to the late stage (41, 56). Seasonal climate change represents an important factor that contributes to the observed variations of the EM fungal community. EM fungal composition and community structure are filtered and shaped by the climate in different forests. Changes in EM fungal community structure affected by the growing phase may have consequences for ecosystem function. On a global scale, EM fungal richness and diversity are primarily influenced by the host plants and environmental factors, such as temperature, precipitation, and substrate quality, which represent energy and water availability, respectively (6, 24). In addition, soil moisture in desert regions is chronically low, so the slight changes in precipitation have a significant impact on the EM fungal community. In summary, the variations in dominant components of the EM fungal communities could be explained by the combined influence of meteorological factors and host status.

EM fungal composition is strongly influenced by biotic and abiotic conditions. To explore the most diverse EM fungal communities associated with Mongolian pine, the sampling times are chosen mainly based on plant phenology. Variations in bio-climate may account for the observed differences in EM fungal community structures between different growing stages. Plant phenology phenomena caused by seasonal climate changes trigger the difference in nutrient requirements in Mongolian pine plantations, which directly or indirectly affects EM fungal composition. EM fungal composition is strongly influenced by biotic and abiotic conditions. In desert ecosystems, high temperatures and drought stresses restrict plant activity and reduce the C allocation to the fungal symbiont, and thus EM fungal diversity and structure are highly vulnerable under such an unfriendly condition. In addition, climatic factors can have a direct influence on the fungal communities as well as indirectly via the plant partner of the symbiosis or changes in the soil conditions. Several meteorological variables were indeed significantly reflected in the EM fungal structure, indicating that Mongolian pine-associated EM fungal communities are susceptible to climate. This finding is not new, as many reports describe the effect of several meteorological drivers on EM fungal communities (6, 57). Moreover, previous studies have shown that precipitation is the main driving factor behind EM fungal community assembly in desert regions (34, 58). The evidence we provide also shows that low water availability during the months of spring and autumn in desert forest ecosystems could be a significant limiting factor of EM fungal survival and diversity, which highlights the importance of fungal responses to climate. These results suggest that climate is the main driver for EM fungal occurrence in desert regions, especially for precipitation. However, the interaction between EM fungi and Mongolian pine is difficult to determine accurately and clearly. EM fungi are driven by the stand age and phenology of the host, so environment conditions play an important role in establishment and survival of the EM fungal community.

### Conclusion

In the Mu Us Desert, the EM fungal community of Mongolian pine is very dynamic, showing significant temporal changes in the relative abundance of EM fungi. We demonstrated that seasonality and the underlying climatic variables affect the diversity and composition of EM fungi. The significant interplay between tree phenology, stand age, and EM fungal community was confirmed. It is worth noting that phenology (seasonal variation) was the most important predictor of EM fungal community than that of stand age (inter-annual variation). The major meteorological elements are mean annual temperature and mean annual precipitation. The relationship between the EM fungal community and the physiology of Mongolian pine plantations changed significantly with tree health status.

## MATERIALS AND METHODS

### Study site

The sampling site was in the Hongshixia Sandy Botanical Garden (N38°19′49″−38°20′12″, E109°42′8″−109°42′55″) of Yulin City, Shaanxi Province, China. The site location is on the southern edge of the Mu Us Desert, with an altitude of 1,080 m. The climate of the study area is defined as a mid-temperate semi-arid continental monsoon climate. The annual temperature is 9.1°C, the average sunshine is 2,914 h, the evaporation is 2,502 mm each year, and annual precipitation is about 385 mm (mainly falling from July to September, accounting for about 70% of the total annual rainfall). The soil consists mainly of eolian sandy soil, which is highly erodible. The arbor plants that dominated this area include *Pinus sylvestris* var. *mongolica*, *Caragana korshinskii,* and *P. tabuliformis*. The dominant shrubs are *Lespedeza bicolor*, *Cares duriuscula*, *Rhamnus parvifolia*, *Salsola collina,* and *Potentilla anserine*.

### Sample collection

Three permanent sampling plots (each 50 × 50 m) of Mongolian pine plantations across three different age groups (27, 34, and 44 yr) were randomly established within the study site, which referred to as MUh (half-mature), MUn (nearly mature), and MUm (mature), respectively. The basic information is summarized in Table 2. Fine root samples were monthly collected in total six times per month from April to 2018 September in total. Sampling dates were chosen along phenology: (i) the middle of April—beginning of growth, (ii) the middle of May—stage of leaf flush, (iii) the middle of June—early growing season, (iv) late July—the peak of the growth season, (v) late August—stage of fruit development, and (vi) late September—leaf abscission. We collected root samples from five individual standard trees in each plot, spaced at least 10 m apart. After removing the loose litter, herbs, and undergrowth humus layer, the terminal root samples were carefully excavated along the base of the trunk. Three repeated samples were taken at each tree followed by a fully mixed root sample of five individual trees. Fine root samples were accompanied with soil to keep the root tips fresh, placed into a sealed bag, and refrigerated at −4°C. A total of 90 mixed root samples were used for DNA extraction and molecular identification of the EM fungi.

**TABLE 2:** General information of different age groups^*a*^

Age group	Stand age/a	Slop/°	Mean height/m	Mean DBH/m	Canopy density
MUh	27	3	12.72 ± 2.56	12.54 ± 2.48	0.79
MUn	33	4	14.06 ± 2.08	13.58 ± 3.44	0.86
MUm	44	4	14.21 ± 2.54	14.92 ± 3.52	0.73

^*a*^ Values are mean ± standard error. DHB, diameter at breast height; MUh, half-mature forest; MUn, nearly mature forest; MUm, mature forest.

### Molecular characterization of EM fungi

The DNA was extracted from fine root samples and purified using the Powersoil DNA Isolation Kit (MoBio, Carlsbad, CA, USA). DNA concentration was quantified on a NanoDrop spectrophotometer (Thermo Scientific, USA). EMF ITS regions were amplified using the common fungal primers ITS1F (5-GGAAGTAAAAGTCGTAACAAGG-3) and ITS2 (5-TCCTCCGCTTATTGATATGC-3). The PCR mixtures were as follows: 4 µL of 5× FastPfu Buffer, 1 µL of each primer (5 µM), 2 µL of dNTP mixture (2.5 mM), 2 µL of template DNA, and 10 µL of H_2_O. The PCR amplification program consisted of an initial denaturation at 95°C for 2 min, followed by 30 cycles of 95°C for 30 s, 55°C for 30 s, 72°C for 30 s, and a final extension at 72°C for 5 min. PCR amplification was performed in triplicate to account for potentially heterogeneous amplification from the environmental template for each sample. PCR products were purified using an AXYGEN Gel Extraction Kit (QIAGEN, Germany). An equimolar mix of all three amplicon libraries was used for sequencing at Allwegene Technology Inc. China, using an Illumina MiSeq sequencing system (Illumina, USA).

### Sequencing data analysis

The raw sequence reads were initially trimmed using the Mothur (Version 1.30.), and sequences that met all three criteria were kept: (i) the sequence with precise primers and barcodes; (ii) quality score >30; (iii) and the sequences >200 bp in length. The software package Usearch was then used to further filter out sequences that were erroneous and chimeric. The remaining high-quality sequences were queried against the GenBank non-redundant nucleotide database (nt) in NCBI using local BLASTn (http://blast.ncbi.nlm.nih.gov/Blast.cgi). The MEGAN program was used to assign BLAST hits to taxa of the NCBI taxonomy. After removing non-fungal sequence reads, the fungal sequences were clustered into OTUs at a 97% similarity level using Uclust. To standardize the depth of different sequencing, the EM fungal OTUs were rarified to the smallest sample size, 4,023.

### Meteorological data analysis

Meteorological data from April to September 2018 were obtained from the China Meteorological Information Date Service Center (CMDC, http://cdc.cma.gov.cn, Table S2). The data we use include sunshine duration, mean minimal temperature, mean maximum temperature, mean temperature, precipitation, rainy days, mean air pressure, mean water pressure, and mean relative humidity.

### Statistical analysis

To the convenience of description, EM fungal genera are classified into dominant genera (>10%), common genera (1%–10%), and rare genera (<1.00%), according to their relative abundance. Based on the genera abundance data, the relative abundance diagram of the EM fungal community composition was drawn with OriginPro 2018.

EM fungal community diversity was measured by Richness, Shannon, Simpson, and Pielou indices and was used to characterize monthly EM fungal community across three age groups. Two-way analysis of variance (ANOVA) followed by multiple comparison tests (least significant difference) was carried out to test for differences in diversity indices among the month and stand age using SPSS 19.0. Results were validated as statistically significant at *P* < 0.05.

NMDS ordination and permutation tests for multivariate analysis of similarity (PERMANOVA) were used to visually observe the differences in EM fungal community structure, based on Bray-Curtis dissimilarity indexes of square-transformed results. All meteorological datum from six sampling months was converted to the matrix to perform the Mantel test. The analysis of variance using distance matrices (ADONIS) and analysis of similarities (ANOSIM) were performed to estimate the significant differences in fungal community composition, with *P* < 0.05 considered significant. NMDS, PERMANOVA, ADONIS, ANOSIM, and Mantel tests were performed with the “vegan” package in R (version 3.5.1).

EM fungal network analyses were performed using the Molecular Ecological Network Analyses Pipeline (http://ieg4.rccc.ou.edu/mena/main.cgi) (59, 60). We analyzed EM fungal network for each stage of the growing season across three age groups separately. To reduce the complexity and ensure the accuracy of data, only kept the OTUs with >10 occurrences. After that, the correlations between pairwise OTUs were constructed based on Pearson correlation, keeping the strong significant correlations with *P* < 0.01 and threshold *r* >0.8. Next, the visualization of the EM fungal network was carried out in Gephi (Version 0.9.2). Network topologies, including modularity, average degree, average path length, average clustering coefficient, and the number of interactions between OTUs, were calculated to demonstrate network structure (57). Key EM fungal species were analyzed using the analysis of modular topological roles (within-module connectivity, *Zi* and among-module connectivity, *Pi*). Network hubs (*Zi* >2.5, *Pi* >0.62), connectors (*Zi* ≤2.5, *Pi* >0.62), and module hubs (*Zi* >2.5, *Pi* ≤0.62) were defined as keystones.

## Data Availability

Raw sequence data have been deposited at the NCBI sequence read archive with accession number PRJNA639377. Details can be found in Table S1.

## AFTER EPUB

*[This article was published on 14 September 2023 with errors in the Acknowledgments and funding table. The errors were corrected in the current version, posted on 21 September 2023.]*

## SUPPLEMENTAL MATERIAL

Fig. S1 and S2 and Tables S1 to S5 spectrum.02026-23-s0001.docx Supplemental figures and tables.

Table S1 spectrum.02026-23-s0002.xlsx Supplemental table.

ASM does not own the copyrights to Supplemental Material that may be linked to, or accessed through, an article. The authors have granted ASM a non-exclusive, world-wide license to publish the Supplemental Material files. Please contact the corresponding author directly for reuse.
