# Supplementary material for: Temporal approach to identifying ectomycorrhizal community associated with Mongolian pine in a desert environment, northern China
Source: Microbiol Spectr. 2023 Sep 14;11(5):e02026-23. doi: 10.1128/spectrum.02026-23 (PMC10580992; doi:10.1128/spectrum.02026-23)
Supplement: Fig. S1 and S2 and Tables S1 to S5 — Supplemental figures and tables. [file spectrum.02026-23-s0001.docx]

Table S2 Climate data of sampling month in 2018

|  | Apr. | May. | Jun. | Jul. | Aug. | Sep. |
| --- | --- | --- | --- | --- | --- | --- |
| Sunshine duration / h | 276.8 | 284.5 | 271.1 | 207.6 | 226.3 | 190.7 |
| Mean minimal temperature / °C | 7.5 | 11.9 | 16.2 | 19.8 | 19 | 10.1 |
| Mean maximum temperature / °C | 20.7 | 24.6 | 28.9 | 28.3 | 28.2 | 19.6 |
| Mean temperature / °C | 13.7 | 18.1 | 22.3 | 23.6 | 22.9 | 14.1 |
| Precipitation / mm | 34.7 | 68.1 | 22.5 | 206.1 | 246.4 | 66.3 |
| Rainy days / d | 7 | 10 | 7 | 12 | 15 | 12 |
| Mean air pressure / hPa | 883.5 | 881.6 | 878.9 | 878.1 | 880.8 | 887 |
| Mean water pressure / hPa | 4.3 | 6.6 | 9.2 | 18 | 17.7 | 9.3 |
| Mean relative humidity / % | 29.5 | 35.2 | 37.1 | 63.5 | 64.8 | 58.1 |

| (a) | 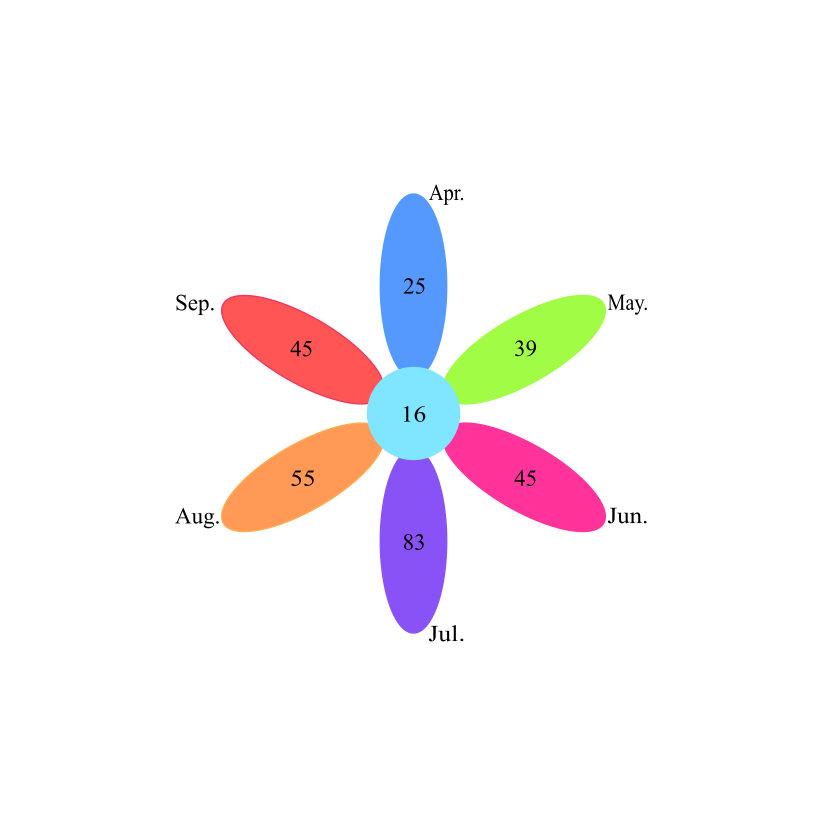 | (b) | 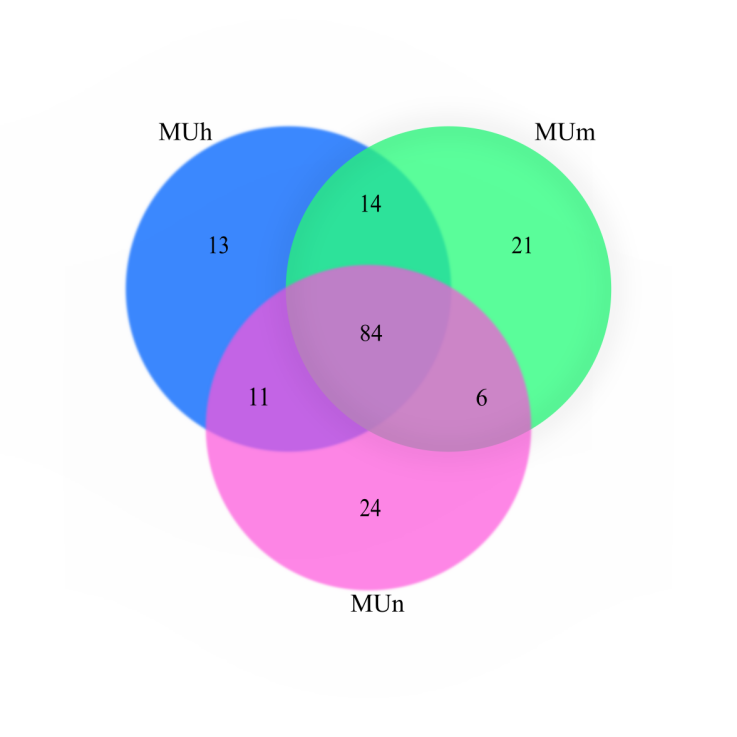 |
| --- | --- | --- | --- |
| Fig. S1 Flower and venn diagram of EM fungal OTUs associated with Mongolian pine at different stages of growing season (a) across three stand ages (b). MUh: half-mature, MUn: nearly-mature, MUm: mature. | | | |


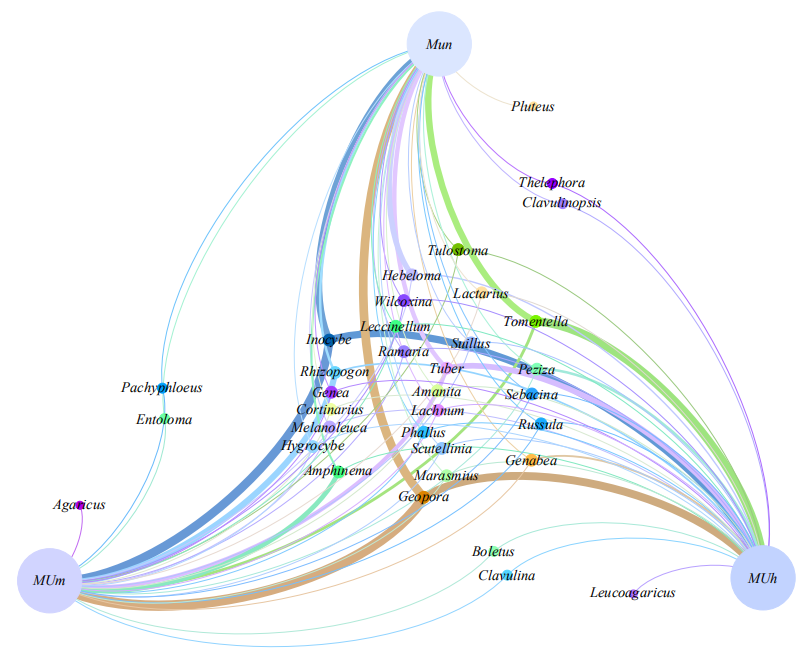


Fig. S2. Network of the EM fungal genera associated with Mongolian pine at different stages of growing season across three stand age. Line thickness represents relative abundance.

Table S3 Alpha diversity of EM fungi in different months and stand ages. Values are mean ± standard error. Same minuscule alphabet in the column indicate non-significant divergence. ^**^: *p* < 0.01; ^*^: *p* < 0.05.

|  |  | Richness | Shannon | Simpson | Pielou |
| --- | --- | --- | --- | --- | --- |
| Month | Apr. | 15.20±3.60 d | 1.67±0.69 c | 0.52±0.21 b | 0.41±0.16 b |
|  | May. | 24.33±3.18 c | 2.33±0.70 b | 0.66±0.17 ab | 0.50±0.1b |
|  | Jun. | 29.20±5.51 b | 3.06±0.54 a | 0.80±0.08 a | 0.63±0.09 a |
|  | Jul. | 35.73±5.44 a | 3.26±0.57 a | 0.80±0.10 a | 0.64±0.11 a |
|  | Aug. | 25.67±4.29 c | 2.82±0.88 a | 0.74±0.22 a | 0.60±0.18 a |
|  | Sep. | 24.73±4.67 c | 2.48±0.65 ab | 0.70±0.12 a | 0.54±0.12 a |
|  | *F* | 30.173^**^ | 9.579^**^ | 5.710^**^ | 4.969^**^ |
| Stand age | MUh | 26.73±7.99 a | 2.72±0.81 a | 0.71±0.16 a | 0.58±0.15 a |
|  | MUn | 24.87±7.32 a | 2.47±0.90 a | 0.68±0.21 a | 0.53±0.17 a |
|  | MUm | 26.33±6.81 a | 2.62±0.83 a | 0.71±0.19 a | 0.55±0.15 a |
|  | *F* | 1.018 | 1.136 | 0.519 | 1.020 |
| Month *Stand age | *F* | 16.268^**^ | 5.384^*^ | 3.267^*^ | 1.003 |

Table S4 Effects of stand age and temporal on the EM fungal community composition. Significant analysis based on 999 times permutation test; bold *p* values indicate significant difference (*p* < 0.05).

| Factor | ADONIS | | ANOSIM | |
| --- | --- | --- | --- | --- |
|  | *R^2^* | *p* | *R* | *p* |
| Stand age | 0.084 | 0.002 | 0.073 | 0.003 |
| Growing season | 0.312 | 0.001 | 0.356 | 0.001 |

Table S5 Mantel test of the EM fungal community, climate factors, growing season and stand age. The significance of the vectors was tested with 999 permutations.

|  | *r* | *p* |
| --- | --- | --- |
| Climate | 0.534 | 0.003 |
| Mean temperature | 0.331 | 0.021 |
| Precipitation | 0.278 | 0.005 |
| Growing season | 0.202 | 0.023 |
| Stand age | 0.004 | 0.452 |

Table S6 Keystone OTUs of the EM fungal communities at different stages of growing season across three age groups.

| Group | OTUs | Taxonomy |
| --- | --- | --- |
| Apr. | OTU115 | k__Fungi; p__Basidiomycota; c__Agaricomycetes; o__Agaricales; f__Inocybaceae; g__Mallocybe; s__Mallocybe_sp |
|  | OTU119 | k__Fungi; p__Basidiomycota; c__Agaricomycetes; o__Agaricales; f__Inocybaceae; g__Mallocybe; s__Mallocybe_sp |
|  | OTU134 | k__Fungi; p__Basidiomycota; c__Agaricomycetes; o__Agaricales; f__Inocybaceae; g__Mallocybe; s__Mallocybe_sp |
|  | OTU223 | k__Fungi; p__Basidiomycota; c__Agaricomycetes; o__Agaricales; f__Inocybaceae; g__Inocybe; s__Inocybe_sp |
|  | OTU324 | k__Fungi; p__Basidiomycota; c__Agaricomycetes; o__Agaricales; f__Hymenogastraceae; g__Hebeloma; s__Hebeloma_mesophaeum |
|  | OTU878 | k__Fungi; p__Basidiomycota; c__Agaricomycetes; o__Thelephorales; f__Thelephoraceae; g__Tomentella; s__Tomentella_sp |
| May. | OTU187 | k__Fungi; p__Basidiomycota; c__Agaricomycetes; o__Agaricales; f__Inocybaceae; g__Inocybe; s__Inocybe_sp |
|  | OTU193 | k__Fungi; p__Basidiomycota; c__Agaricomycetes; o__Agaricales; f__Inocybaceae; g__Inocybe; s__Inocybe_sp |
|  | OTU254 | k__Fungi; p__Basidiomycota; c__Agaricomycetes; o__Agaricales; f__Inocybaceae; g__Inocybe; s__Inocybe_hygrophana |
|  | OTU510 | k__Fungi; p__Basidiomycota; c__Agaricomycetes; o__Agaricales; f__Inocybaceae; g__Inocybe; s__Inocybe_sp |
|  | OTU324 | k__Fungi; p__Basidiomycota; c__Agaricomycetes; o__Agaricales; f__Hymenogastraceae; g__Hebeloma; s__Hebeloma_mesophaeum |
|  | OTU324 | k__Fungi; p__Basidiomycota; c__Agaricomycetes; o__Agaricales; f__Hymenogastraceae; g__Hebeloma; s__Hebeloma_mesophaeum |
|  | OTU838 | k__Fungi; p__Basidiomycota; c__Agaricomycetes; o__Thelephorales; f__Thelephoraceae; g__Tomentella; s__Tomentella_sp |
|  | OTU652 | k__Fungi; p__Ascomycota; c__Pezizomycetes; o__Pezizales; f__Pyronemataceae; g__Geopora; s__Geopora_sp |
|  | OTU683 | k__Fungi; p__Ascomycota; c__Pezizomycetes; o__Pezizales; f__Pyronemataceae; g__Geopora; s__Geopora_arenicola |
| Jun. | OTU119 | k__Fungi; p__Basidiomycota; c__Agaricomycetes; o__Agaricales; f__Inocybaceae; g__Mallocybe; s__Mallocybe_sp |
|  | OTU226 | k__Fungi; p__Basidiomycota; c__Agaricomycetes; o__Agaricales; f__Inocybaceae; g__Inocybe; s__Inocybe_subporospora |
|  | OTU257 | k__Fungi; p__Basidiomycota; c__Agaricomycetes; o__Agaricales; f__Inocybaceae; g__Inocybe; s__Inocybe_abjecta |
|  | OTU353 | k__Fungi; p__Basidiomycota; c__Agaricomycetes; o__Agaricales; f__Hymenogastraceae; g__Hebeloma; s__Hebeloma_salicicola |
|  | OTU470 | k__Fungi; p__Basidiomycota; c__Agaricomycetes; o__Agaricales; f__Inocybaceae; g__Inocybe; s__Inocybe_sp |
|  | OTU878 | k__Fungi; p__Basidiomycota; c__Agaricomycetes; o__Thelephorales; f__Thelephoraceae; g__Tomentella; s__Tomentella_sp |
|  | OTU880 | k__Fungi; p__Basidiomycota; c__Agaricomycetes; o__Thelephorales; f__Thelephoraceae; g__Tomentella; s__Tomentella_sp |
|  | OTU458 | k__Fungi; p__Ascomycota; c__Pezizomycetes; o__Pezizales; f__Pyronemataceae; g__Scutellinia; s__Scutellinia_sp |
|  | OTU561 | k__Fungi; p__Ascomycota; c__Pezizomycetes; o__Pezizales; f__Pyronemataceae; g__Geopora; s__Geopora_pinyonensis |
|  | OTU652 | k__Fungi; p__Ascomycota; c__Pezizomycetes; o__Pezizales; f__Pyronemataceae; g__Geopora; s__Geopora_sp |
|  | OTU680 | k__Fungi; p__Ascomycota; c__Pezizomycetes; o__Pezizales; f__Pyronemataceae; g__Geopora; s__Geopora_arenicola |
|  | OTU915 | k__Fungi; p__Ascomycota; c__Pezizomycetes; o__Pezizales; f__Tuberaceae; g__Tuber; s__Tuber_oligospermum |
| Jul. | OTU1427 | k__Fungi; p__Basidiomycota; c__Agaricomycetes; o__Atheliales; f__Atheliaceae; g__Amphinema; s__Amphinema_sp |
|  | OTU1677 | k__Fungi; p__Basidiomycota; c__Agaricomycetes; o__Atheliales; f__Atheliaceae; g__Amphinema; s__Amphinema_sp |
|  | OTU147 | k__Fungi; p__Basidiomycota; c__Agaricomycetes; o__Agaricales; f__Inocybaceae; g__Mallocybe; s__Mallocybe_sp |
|  | OTU865 | k__Fungi; p__Basidiomycota; c__Agaricomycetes; o__Thelephorales; f__Thelephoraceae; g__Tomentella; s__Tomentella_sp |
|  | OTU2032 | k__Fungi; p__Ascomycota; c__Pezizomycetes; o__Pezizales; f__Pyronemataceae; g__Genabea; s__Genabea_fragilis |
|  | OTU683 | k__Fungi; p__Ascomycota; c__Pezizomycetes; o__Pezizales; f__Pyronemataceae; g__Geopora; s__Geopora_arenicola |
|  | OTU915 | k__Fungi; p__Ascomycota; c__Pezizomycetes; o__Pezizales; f__Tuberaceae; g__Tuber; s__Tuber_oligospermum |
| Aug. | OTU115 | k__Fungi; p__Basidiomycota; c__Agaricomycetes; o__Agaricales; f__Inocybaceae; g__Mallocybe; s__Mallocybe_sp |
|  | OTU1274 | k__Fungi; p__Basidiomycota; c__Agaricomycetes; o__Russulales; f__Lachnocladiaceae; g__Asterostroma; s__Asterostroma_cervicolor |
|  | OTU134 | k__Fungi; p__Basidiomycota; c__Agaricomycetes; o__Agaricales; f__Inocybaceae; g__Mallocybe; s__Mallocybe_sp |
|  | OTU187 | k__Fungi; p__Basidiomycota; c__Agaricomycetes; o__Agaricales; f__Inocybaceae; g__Inocybe; s__Inocybe_sp |
|  | OTU193 | k__Fungi; p__Basidiomycota; c__Agaricomycetes; o__Agaricales; f__Inocybaceae; g__Inocybe; s__Inocybe_sp |
|  | OTU223 | k__Fungi; p__Basidiomycota; c__Agaricomycetes; o__Agaricales; f__Inocybaceae; g__Inocybe; s__Inocybe_sp |
|  | OTU324 | k__Fungi; p__Basidiomycota; c__Agaricomycetes; o__Agaricales; f__Hymenogastraceae; g__Hebeloma; s__Hebeloma_mesophaeum |
|  | OTU449 | k__Fungi; p__Basidiomycota; c__Agaricomycetes; o__Agaricales; f__Marasmiaceae; g__Marasmius; s__Marasmius_sullivantii |
|  | OTU856 | k__Fungi; p__Basidiomycota; c__Agaricomycetes; o__Thelephorales; f__Thelephoraceae; g__Tomentella; s__Tomentella_sp |
|  | OTU458 | k__Fungi; p__Ascomycota; c__Pezizomycetes; o__Pezizales; f__Pyronemataceae; g__Scutellinia; s__Scutellinia_sp |
|  | OTU561 | k__Fungi; p__Ascomycota; c__Pezizomycetes; o__Pezizales; f__Pyronemataceae; g__Geopora; s__Geopora_pinyonensis |
|  | OTU623 | k__Fungi; p__Ascomycota; c__Pezizomycetes; o__Pezizales; f__Pyronemataceae; g__Geopora; s__Geopora_sp |
|  | OTU683 | k__Fungi; p__Ascomycota; c__Pezizomycetes; o__Pezizales; f__Pyronemataceae; g__Geopora; s__Geopora_arenicola |
| Sep. | OTU134 | k__Fungi; p__Basidiomycota; c__Agaricomycetes; o__Agaricales; f__Inocybaceae; g__Mallocybe; s__Mallocybe_sp |
|  | OTU193 | k__Fungi; p__Basidiomycota; c__Agaricomycetes; o__Agaricales; f__Inocybaceae; g__Inocybe; s__Inocybe_sp |
|  | OTU317 | k__Fungi; p__Basidiomycota; c__Agaricomycetes; o__Agaricales; f__Tricholomataceae; g__Tricholoma; s__Tricholoma_terreum |
|  | OTU324 | k__Fungi; p__Basidiomycota; c__Agaricomycetes; o__Agaricales; f__Hymenogastraceae; g__Hebeloma; s__Hebeloma_mesophaeum |
|  | OTU793 | k__Fungi; p__Basidiomycota; c__Agaricomycetes; o__Thelephorales; f__Thelephoraceae; g__Tomentella; s__Tomentella_sp |
|  | OTU856 | k__Fungi; p__Basidiomycota; c__Agaricomycetes; o__Thelephorales; f__Thelephoraceae; g__Tomentella; s__Tomentella_sp |
|  | OTU623 | k__Fungi; p__Ascomycota; c__Pezizomycetes; o__Pezizales; f__Pyronemataceae; g__Geopora; s__Geopora_sp |
|  | OTU680 | k__Fungi; p__Ascomycota; c__Pezizomycetes; o__Pezizales; f__Pyronemataceae; g__Geopora; s__Geopora_arenicola |
|  | OTU683 | k__Fungi; p__Ascomycota; c__Pezizomycetes; o__Pezizales; f__Pyronemataceae; g__Geopora; s__Geopora_arenicola |
| MUh | OTU1427 | k__Fungi; p__Basidiomycota; c__Agaricomycetes; o__Atheliales; f__Atheliaceae; g__Amphinema; s__Amphinema_sp |
|  | OTU1677 | k__Fungi; p__Basidiomycota; c__Agaricomycetes; o__Atheliales; f__Atheliaceae; g__Amphinema; s__Amphinema_sp |
|  | OTU1754 | k__Fungi; p__Ascomycota; c__Pezizomycetes; o__Pezizales; f__Pezizaceae; g__Delastria; s__Delastria_sp |
|  | OTU187 | k__Fungi; p__Basidiomycota; c__Agaricomycetes; o__Agaricales; f__Inocybaceae; g__Inocybe; s__Inocybe_sp |
|  | OTU193 | k__Fungi; p__Basidiomycota; c__Agaricomycetes; o__Agaricales; f__Inocybaceae; g__Inocybe; s__Inocybe_sp |
|  | OTU448 | k__Fungi; p__Basidiomycota; c__Agaricomycetes; o__Agaricales; f__Inocybaceae; g__Inocybe; s__Inocybe_exilis |
|  | OTU470 | k__Fungi; p__Basidiomycota; c__Agaricomycetes; o__Agaricales; f__Inocybaceae; g__Inocybe; s__Inocybe_sp |
|  | OTU510 | k__Fungi; p__Basidiomycota; c__Agaricomycetes; o__Agaricales; f__Inocybaceae; g__Inocybe; s__Inocybe_sp |
|  | OTU719 | k__Fungi; p__Basidiomycota; c__Agaricomycetes; o__Agaricales; f__Inocybaceae; g__Inocybe; s__Inocybe_pruinosa |
|  | OTU477 | k__Fungi; p__Basidiomycota; c__Agaricomycetes; o__Boletales; f__Rhizopogonaceae; g__Rhizopogon; s__Rhizopogon_sp |
|  | OTU793 | k__Fungi; p__Basidiomycota; c__Agaricomycetes; o__Thelephorales; f__Thelephoraceae; g__Tomentella; s__Tomentella_sp |
|  | OTU853 | k__Fungi; p__Basidiomycota; c__Agaricomycetes; o__Thelephorales; f__Thelephoraceae; g__Tomentella; s__Tomentella_sp |
|  | OTU878 | k__Fungi; p__Basidiomycota; c__Agaricomycetes; o__Thelephorales; f__Thelephoraceae; g__Tomentella; s__Tomentella_sp |
|  | OTU901 | k__Fungi; p__Basidiomycota; c__Agaricomycetes; o__Thelephorales; f__Thelephoraceae; g__Tomentella; s__Tomentella_sp |
|  | OTU561 | k__Fungi; p__Ascomycota; c__Pezizomycetes; o__Pezizales; f__Pyronemataceae; g__Geopora; s__Geopora_pinyonensis |
|  | OTU652 | k__Fungi; p__Ascomycota; c__Pezizomycetes; o__Pezizales; f__Pyronemataceae; g__Geopora; s__Geopora_sp |
| MUn | OTU115 | k__Fungi; p__Basidiomycota; c__Agaricomycetes; o__Agaricales; f__Inocybaceae; g__Mallocybe; s__Mallocybe_sp |
|  | OTU119 | k__Fungi; p__Basidiomycota; c__Agaricomycetes; o__Agaricales; f__Inocybaceae; g__Mallocybe; s__Mallocybe_sp |
|  | OTU125 | k__Fungi; p__Basidiomycota; c__Agaricomycetes; o__Agaricales; f__Inocybaceae; g__Mallocybe; s__Mallocybe_sp |
|  | OTU1427 | k__Fungi; p__Basidiomycota; c__Agaricomycetes; o__Atheliales; f__Atheliaceae; g__Amphinema; s__Amphinema_sp |
|  | OTU1677 | k__Fungi; p__Basidiomycota; c__Agaricomycetes; o__Atheliales; f__Atheliaceae; g__Amphinema; s__Amphinema_sp |
|  | OTU147 | k__Fungi; p__Basidiomycota; c__Agaricomycetes; o__Agaricales; f__Inocybaceae; g__Mallocybe; s__Mallocybe_sp |
|  | OTU187 | k__Fungi; p__Basidiomycota; c__Agaricomycetes; o__Agaricales; f__Inocybaceae; g__Inocybe; s__Inocybe_sp |
|  | OTU193 | k__Fungi; p__Basidiomycota; c__Agaricomycetes; o__Agaricales; f__Inocybaceae; g__Inocybe; s__Inocybe_sp |
|  | OTU461 | k__Fungi; p__Basidiomycota; c__Agaricomycetes; o__Agaricales; f__Inocybaceae; g__Inocybe; s__Inocybe_sp |
|  | OTU507 | k__Fungi; p__Basidiomycota; c__Agaricomycetes; o__Agaricales; f__Inocybaceae; g__Inocybe; s__Inocybe_dunensis |
|  | OTU510 | k__Fungi; p__Basidiomycota; c__Agaricomycetes; o__Agaricales; f__Inocybaceae; g__Inocybe; s__Inocybe_sp |
|  | OTU475 | k__Fungi; p__Basidiomycota; c__Agaricomycetes; o__Boletales; f__Rhizopogonaceae; g__Rhizopogon; s__Rhizopogon_jiyaozi |
|  | OTU477 | k__Fungi; p__Basidiomycota; c__Agaricomycetes; o__Boletales; f__Rhizopogonaceae; g__Rhizopogon; s__Rhizopogon_sp |
|  | OTU317 | k__Fungi; p__Basidiomycota; c__Agaricomycetes; o__Agaricales; f__Tricholomataceae; g__Tricholoma; s__Tricholoma_terreum |
|  | OTU324 | k__Fungi; p__Basidiomycota; c__Agaricomycetes; o__Agaricales; f__Hymenogastraceae; g__Hebeloma; s__Hebeloma_mesophaeum |
|  | OTU353 | k__Fungi; p__Basidiomycota; c__Agaricomycetes; o__Agaricales; f__Hymenogastraceae; g__Hebeloma; s__Hebeloma_salicicola |
|  | OTU461 | k__Fungi; p__Basidiomycota; c__Agaricomycetes; o__Agaricales; f__Inocybaceae; g__Inocybe; s__Inocybe_sp |
|  | OTU2032 | k__Fungi; p__Ascomycota; c__Pezizomycetes; o__Pezizales; f__Pyronemataceae; g__Genabea; s__Genabea_fragilis |
|  | OTU574 | k__Fungi; p__Ascomycota; c__Pezizomycetes; o__Pezizales; f__Pyronemataceae; g__Geopora; s__Geopora_sp |
| MUm | OTU119 | k__Fungi; p__Basidiomycota; c__Agaricomycetes; o__Agaricales; f__Inocybaceae; g__Mallocybe; s__Mallocybe_sp |
|  | OTU1274 | k__Fungi; p__Basidiomycota; c__Agaricomycetes; o__Russulales; f__Lachnocladiaceae; g__Asterostroma; s__Asterostroma_cervicolor |
|  | OTU134 | k__Fungi; p__Basidiomycota; c__Agaricomycetes; o__Agaricales; f__Inocybaceae; g__Mallocybe; s__Mallocybe_sp |
|  | OTU1754 | k__Fungi; p__Ascomycota; c__Pezizomycetes; o__Pezizales; f__Pezizaceae; g__Delastria; s__Delastria_sp |
|  | OTU187 | k__Fungi; p__Basidiomycota; c__Agaricomycetes; o__Agaricales; f__Inocybaceae; g__Inocybe; s__Inocybe_sp |
|  | OTU193 | k__Fungi; p__Basidiomycota; c__Agaricomycetes; o__Agaricales; f__Inocybaceae; g__Inocybe; s__Inocybe_sp |
|  | OTU480 | k__Fungi; p__Basidiomycota; c__Agaricomycetes; o__Agaricales; f__Inocybaceae; g__Inocybe; s__Inocybe_sp |
|  | OTU507 | k__Fungi; p__Basidiomycota; c__Agaricomycetes; o__Agaricales; f__Inocybaceae; g__Inocybe; s__Inocybe_dunensis |
|  | OTU353 | k__Fungi; p__Basidiomycota; c__Agaricomycetes; o__Agaricales; f__Hymenogastraceae; g__Hebeloma; s__Hebeloma_salicicola |
|  | OTU475 | k__Fungi; p__Basidiomycota; c__Agaricomycetes; o__Boletales; f__Rhizopogonaceae; g__Rhizopogon; s__Rhizopogon_jiyaozi |
|  | OTU865 | k__Fungi; p__Basidiomycota; c__Agaricomycetes; o__Thelephorales; f__Thelephoraceae; g__Tomentella; s__Tomentella_sp |
|  | OTU880 | k__Fungi; p__Basidiomycota; c__Agaricomycetes; o__Thelephorales; f__Thelephoraceae; g__Tomentella; s__Tomentella_sp |
|  | OTU901 | k__Fungi; p__Basidiomycota; c__Agaricomycetes; o__Thelephorales; f__Thelephoraceae; g__Tomentella; s__Tomentella_sp |
|  | OTU2032 | k__Fungi; p__Ascomycota; c__Pezizomycetes; o__Pezizales; f__Pyronemataceae; g__Genabea; s__Genabea_fragilis |
|  | OTU561 | k__Fungi; p__Ascomycota; c__Pezizomycetes; o__Pezizales; f__Pyronemataceae; g__Geopora; s__Geopora_pinyonensis |
|  | OTU550 | k__Fungi; p__Ascomycota; c__Pezizomycetes; o__Pezizales; f__Pyronemataceae; g__Geopora; s__Geopora_sp |
|  | OTU623 | k__Fungi; p__Ascomycota; c__Pezizomycetes; o__Pezizales; f__Pyronemataceae; g__Geopora; s__Geopora_sp |
|  | OTU683 | k__Fungi; p__Ascomycota; c__Pezizomycetes; o__Pezizales; f__Pyronemataceae; g__Geopora; s__Geopora_arenicola |
